# IQGAP1 in Podosomes/Invadosomes Is Involved in the Progression of Glioblastoma Multiforme Depending on the Tumor Status

**DOI:** 10.3390/ijms18010150

**Published:** 2017-01-13

**Authors:** Deborah Rotoli, Natalia Dolores Pérez-Rodríguez, Manuel Morales, María del Carmen Maeso, Julio Ávila, Ali Mobasheri, Pablo Martín-Vasallo

**Affiliations:** 1 Laboratorio de Biología del Desarrollo, UD de Bioquímica y Biología Molecular and Centro de Investigaciones Biomédicas de Canarias (CIBICAN), Universidad de La Laguna, La Laguna, Av. Astrofísico Sánchez s/n, 38206 La Laguna, Tenerife, Spain; deborah_rotoli@yahoo.it (D.R.); javila@ull.es (J.Á.); 2 CNR–National Research Council, Institute of Endocrinology and Experimental Oncology (IEOS), Via Sergio Pansini, 5-80131 Naples, Italy; 3 Service of Medical Oncology, University Hospital Nuestra Señora de Candelaria, 38010 Santa Cruz de Tenerife, Canary Islands, Spain; natalia.perezrodriguez@gmail.com (N.D.P.-R.); mmoraleg@ull.es (M.M.); 4 Medical Oncology, Hospiten^®^ Hospitals, 38001 Santa Cruz de Tenerife, Tenerife, Spain; 5 Service of Pathology, University Hospital Nuestra Señora de Candelaria, 38010 Santa Cruz de Tenerife, Canary Islands, Spain; mmaefor@gmail.com; 6 Faculty of Health and Medical Sciences, University of Surrey, Guildford, Surrey GU2 7XH, UK; a.mobasheri@surrey.ac.uk; 7 Center of Excellence in Genomic Medicine Research (CEGMR), King Fahd Medical Research Center (KFMRC), Faculty of Applied Medical Sciences, King AbdulAziz University, Jeddah 21589, Saudi Arabia

**Keywords:** IQGAP1, glioblastoma multiforme (GBM), scaffold protein, podosome/invadosome

## Abstract

Glioblastoma multiforme (GBM) is the most frequent and aggressive primary brain tumor. GBM is formed by a very heterogeneous astrocyte population, neurons, neovascularization and infiltrating myeloid cells (microglia and monocyte derived macrophages). The IQGAP1 scaffold protein interacts with components of the cytoskeleton, cell adhesion molecules, and several signaling molecules to regulate cell morphology and motility, cell cycle and other cellular functions. IQGAP1 overexpression and delocalization has been observed in several tumors, suggesting a role for this protein in cell proliferation, transformation and invasion. IQGAP1 has been identified as a marker of amplifying cancer cells in GBMs. To determine the involvement of IQGAP1 in the onco-biology of GBM, we performed immunohistochemical confocal microscopic analysis of the IQGAP1 protein in human GBM tissue samples using cell type-specific markers. IQGAP1 immunostaining and subcellular localization was heterogeneous; the protein was located in the plasma membrane and, at variable levels, in nucleus and/or cytosol. Moreover, IQGAP1 positive staining was found in podosome/invadopodia-like structures. IQGAP1^+^ staining was observed in neurons (Map2^+^ cells), in cancer stem cells (CSC; nestin^+^) and in several macrophages (CD31^+^ or Iba1^+^). Our results indicate that the IQGAP1 protein is involved in normal cell physiology as well as oncologic processes.

## 1. Introduction

Glioblastoma multiforme (GBM) is the most frequent and aggressive of all primary brain tumors, characterized by poor outcomes; after surgery and radiotherapy, the median survival of patients is less than one year; the overall survival (OS) at two years is less than 10%; and the OS at five years is less than 2% [1,2,3]. Therefore, exploring the molecular bases of pathogenesis and progression of GBM has important medical value and social significance. Routine molecular biomarkers for diagnosis, predicting prognosis and stratifying patients for therapies now include assessing B-Raf proto-oncogene (BRAF) and isocitrate dehydrogenase 1/2 (IDH1/2) mutations, O-6-methylguanine-DNA methyltransferase (MGMT) promoter methylation as well as predictor of response to temozolomide, a pivotal drug in GBM therapy [2,4,5,6,7].

Despite intra-tumor morphological and molecular heterogeneity, there are processes and molecules common to every GBM cell that could be taken as target for better understanding GBM biology and therapy setting. Invadosome formation and progression is a key process in tumor progression including cell growth, angiogenesis, invasion and metastasis.

IQGAP1 (IQ motif containing GTPase-activating protein 1) is a scaffold multidomain protein ubiquitously expressed. IQGAP1 connects elements of the cytoskeleton to cell adhesion and some signaling molecules and thus modulates cell morphology, motility, cell cycle and other cellular functions [8,9], always facilitating and coordinating the spatiotemporal organization and the sequential activation of structural and signaling molecules [10,11]. Because of this association with molecular partners, IQGAP1 contributes to cell fate, polarization, tumorigenesis, migration, tumor progression and angiogenesis [12,13].

Recent studies have reported that IQGAP1 is accumulated in the plasma membrane at the invasive front in several cancer types [14,15], including gliomas [16]. However, very little is known of IQGAP1 in GBM. PCR or Western blotting to study gene and protein expression in whole tumor pieces does not give a real information of GBM tumorigenesis or progression. The approach that we have taken in this study is double and triple immunolabeling in confocal microscopy in order to achieve a “cell by cell” analysis to obtain high quality subcellular data. Table 1 lists the primary antibodies used in this study as markers of specific cell types; detailed information can be found in Material and Methods.

In this study, we show evidence of IQGAP1 involvement in GBM onco-biology, including glioma-infiltrating myeloid cells (GIMs), remaining neurons, endothelial vascular cells, and astrocytes. Enrichment of IQGAP1 in invadopodia suggests an involvement of IQGAP1 in the invasion process of GBM cells and in podosomes of tumor associated macrophages (TAMs).

## 2. Results

**Figure 1:**
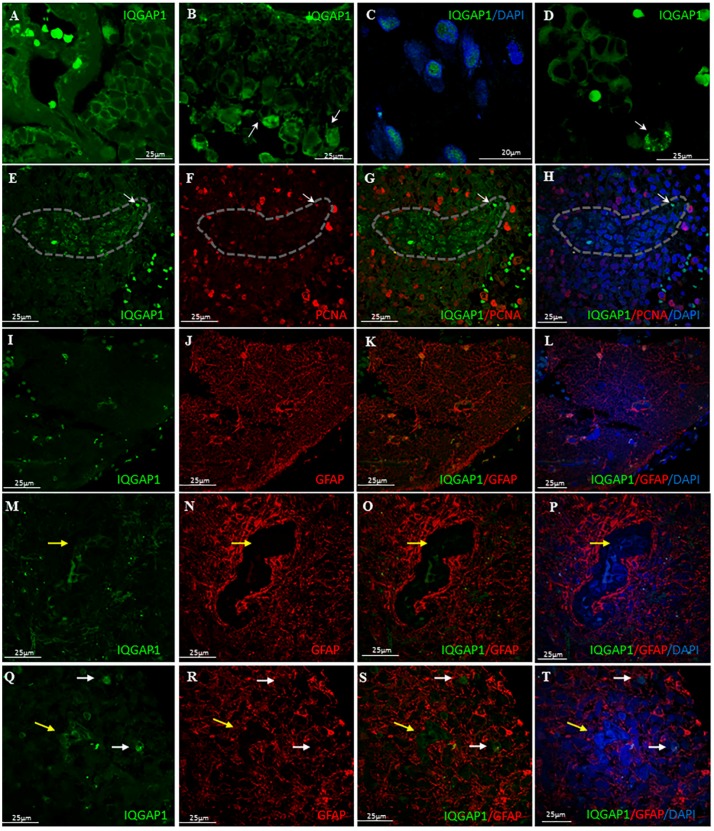
(**A**–**D**) Immunohistochemical characterization of glioblastoma multiforme tumors; Localization of IQGAP1: plasma membrane (**A**); plasma membrane and cytosol (**B**); nuclear (**C**); and cell protrusions (white arrows in **B**). Arrow in (**D**) points to a cell with podosome-like structures highly positive for IQGAP1; (**E**–**H**) GBM double immunostained for IQGAP1 protein (green) and the proliferative marker PCNA (red). Dashed line delimits an IQGAP1^+^ cell cluster that exhibits variable intensities of PCNA immunostaining, but always at a lesser extent than the surrounding cells, where the intensity of PCNA positive signal is high. Thin arrow points to an IQGAP1^+^ tumor-associated microvessel; (**I**–**T**) Double immunolocalization of IQGAP1 protein (green) and the astrocyte marker GFAP (red). (**I**–**L**) Astrocytes (GFAP^+^) are also IQGAP1^+^ at variable intensities; (**M**–**P**) IQGAP1 immunoreactivity is present in a cluster of cells encapsulated by GFAP^+^ cells (yellow arrow), and in microglia; (**Q**–**T**) Frequently, the presence of macrophage-like IQGAP1^+^ cells (white arrows) is observed in areas surrounding the IQGAP1^+^/GFAP^−^ clusters (yellow arrows). To assess cellularity, nuclei were counterstained with DAPI (blue). Bar, 25 μm, Bar in panel **C**, 20 μm.

IQGAP1 immunoreactivity in GBM tissue sections exhibited a variable pattern of expression, depending on the origin of tumor (patient case), area of tumor and even in specific astrocytes within the same tumor. Staining pattern and localization were heterogeneous, with no apparent differences between primary and secondary GBMs. The expression levels varied from moderate to high. In all cases studied, plasma membrane localization of IQGAP1 protein was observed, while the presence of the protein in other compartments (nucleus and/or cytosol) was variable (Figure 1A; Supplementary Martials Figure S1A). In many cells, a strong punctate or ring-shaped staining was observed in cell protrusions (Figure 1B) and in areas adjacent to the nucleus (Figure 1C). Table 2 illustrates variability in IQGAP1 localization and intensity in primary and secondary GBM in selected patients.

When we performed double immunofluorescent experiments to visualize IQGAP1 and PCNA protein expression in GBM tissue sections, we observed clusters of IQGAP1^+^ cells exhibiting variable intensities of PCNA immunostaining, but always at a lesser extent than the surrounding cells, where the intensity of PCNA signal was high (Figure 1E–H).

Double immunostaining analyses of GBM tissue sections labeled for IQGAP1 and for the Glial Fibrillar Acidic Protein (GFAP) revealed that astrocytes (GFAP^+^) were also IQGAP1^+^ at variable intensities (Figure 1I–T).

Clusters of IQGAP1^+^/GFAP^−^ cells were detected surrounded by GFAP^+^ cells (Figure 1M–T, yellow arrow). Frequently, these clusters were located around tumor associated microvessels. Moreover, in neighboring areas of these clusters, the presence of IQGAP1^+^ macrophage-like cells was observed. (Figure 1Q–T, white arrows).

**Figure 2:**
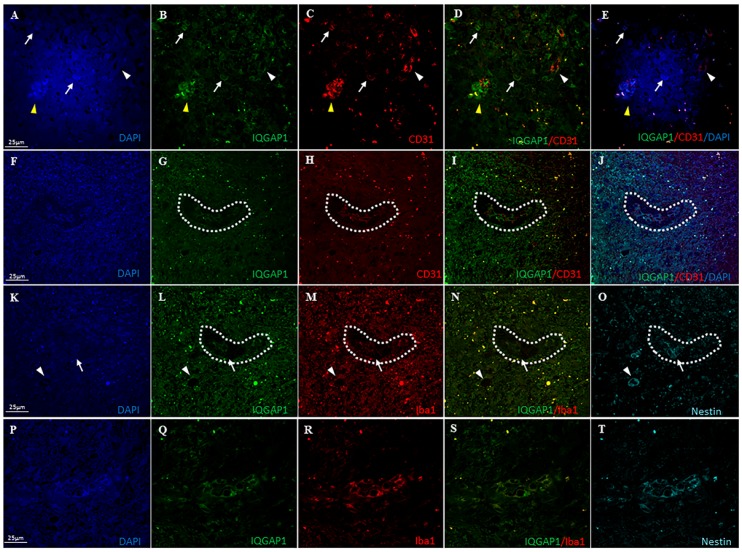
(**A**–**L**) Double immunofluorescent staining of IQGAP1 protein (green) and the pan macrophage and endothelial marker CD31 (red) in human GBM tissue sections. (**A**–**E**) IQGAP1^+^/CD31^+^ macrophages are observed either as isolated cells (white arrows) or as clusters of cells (yellow arrowhead); white arrowhead: blood vessel positive for IQGAP1 and CD31; (**F**–**J**) In IQGAP1^+^ clusters (specified by dashed line), CD31^+^ blood vessels and CD31^+^ cells are observed, while outside the cluster cells are CD31^−^; (**K**–**O**) Triple immunolocalization of IQGAP1 protein (green), Nestin (cyan) and Iba1 (red) in a serial section of the same tissue specimen shown in (**F**–**J**). Cells surrounding IQGAP1^+^ cluster are Iba1^+^. In the cluster, not all of the cells co-express nestin and/or Iba1. Arrow points to a nestin^+^/IQGAP1^−^/Iba1^−^ cell flanked by nestin^+^/IQGAP1^+^/Iba1^+^ cells. Arrowhead points to a nestin^+^ blood vessel; note the presence of IQGAP1^+^/Iba1^+^ cells placed around the vessel; (**P**–**T**) IQGAP1^+^ cluster in which all cells coexpress IQGAP1, Nestin and Iba1. Nuclei were counterstained with DAPI (blue). Bar, 25 μm.

To confirm the TAMs nature of these cells, the macrophage specific marker CD31 was used as a marker for endothelial cells and monocyte-derived macrophages [17] and Iba1 (ionized calcium-binding adapter molecule 1) as a microglia/macrophage marker, commonly used to label total tumor associated macrophagies (TAMs) [18,19,20]. In our experiments, in all tumor-associated blood vessels, a coimmunolocalization of IQGAP1 and CD31 was observed (Figure 2A–E). Moreover, IQGAP1^+^/CD31^+^ macrophages were observed either as isolated cells or as clusters of cells (Figure 2A–E, white arrows and yellow arrowhead, respectively).

To further characterize IQGAP1^+^ cells, the intermediate filament protein nestin was used as a marker of neural stem/progenitor cells in GBM [21,22,23,24,25,26] in combination with Iba1. As can be observed in Figure 2K–O, frequently in nestin^+^ blood vessels, IQGAP1^+^/Iba1^+^ cells were observed in close proximity to the vessel wall (arrowhead). Moreover, in some IQGAP1^+^ clusters, not all of the cells co-expressed nestin and/or Iba1. Indeed, we observed nestin^+^ cells flanked by IQGAP1^+^/Iba1^+^/nestin^+^ cells (Figure 2K–O, arrows; enlarged in Supplementary Materials, Figure S1B). However, in other IQGAP1^+^ clusters, all cells did express the three proteins simultaneously (Figure 2P–T). The origin of glioma-infiltrating myeloid cells (GIMs) surrounding IQGAP1^+^ clusters seem to be from resident brain macrophages (microglia), since cells were Iba1^+^/CD31^−^ (Figure 2H,M).

**Figure 3:**
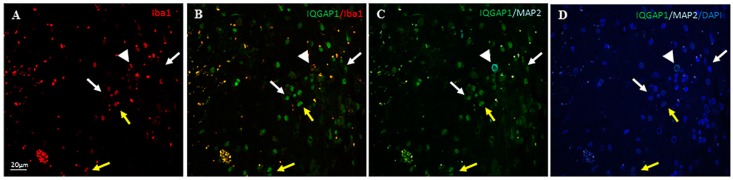

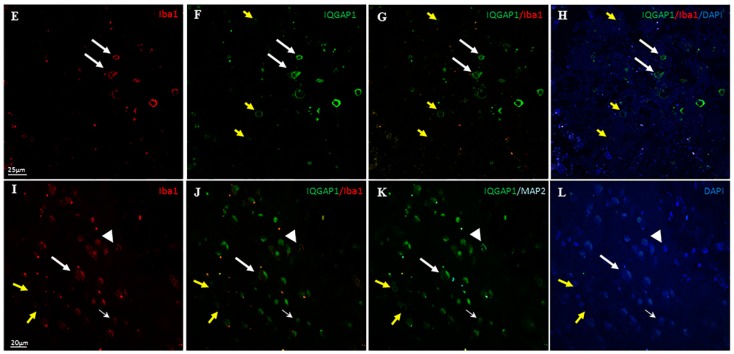
Triple immunolocalization of IQGAP1 protein (green), the microglia/macrophage marker Iba1 (red) and the Microtubule-associated protein 2 (MAP2, cyan) in GBM tissue sections. (**A**–**D**) several Iba1^+^ cells are also IQGAP1^+^ (yellow arrows); white arrows point to IQGAP1^+^/Iba1^−^ cells; arrowhead in (**C**,**D**) points to a neuron Map2^+^/IQGAP1^+^/Iba1^+^; (**E**–**H**) In this area of high micro-vascularization, several macrophages IQGAP1^+^/Iba1^+^ are detected (white arrows); yellow arrows point to microvessels IQGAP1^+^; (**I**–**L**) In Tumor Associated Macrophages (TAMs), both IQGAP1 and Iba1 proteins are enriched and colocalize in a polarized manner close to the nucleus; faint immunolocalization can also be observed in the lamellum at the leading edge of cells (thick arrow). Thin arrow points to a cell where IQGAP1 and Iba1 colocalize in a structure that resembles the actin-ring core of podosomes/invadopodia; arrowhead points to a neuron Map2^+^/IQGAP1^+^/Iba1^+^; yellow arrows point to axons Map2^+^. To assess cellularity, nuclei were counterstained with DAPI (blue). Bar in panels **A**–**D** and **I**–**L**, 20 μm; bar in panels **E**–**H**, 25 μm.

To investigate if the expression of Iba1 and IQGAP1 correlates with the undifferentiated status of the cell, we performed triple immune localization to detect IQGAP1, Iba 1 and the mature neuronal marker Map2 (microtubule associated protein 2) proteins. Immunostaining of IQGAP1 and Iba1 in Map2^+^ neurons was heterogeneous, with neurons expressing both proteins (Figure 3C,D,I–L) or neither (Supplementary Materials, Figure S2).

Regarding IQGAP1^−^Iba1 coexpression experiments, while few IQGAP1^+^ cells did not co-stain with Iba1 (white arrows in Figure 3), many others were positive for Iba1 (Figure 3 yellow arrows in A–D; white arrows in E–H), confirming the macrophage lineage of such cells. Frequently IQGAP1^+^/Iba1^+^ macrophages were detected in areas of high micro-vessel concentration (Figure 3E–H; Supplementary Materials, Figure S3).

In many IQGAP1 and Iba1 colocalizing cells, a more intense staining for these proteins was found and in a polarized manner, close to nuclei, drawing an image resembling the actin-ring core of podosomes/invadopodia (Figure 3I–L, thin arrow). Faint immunocolocalization was also found in lamelli at the leading edge of cells (Figure 3I–L, thick arrow).

**Figure 4:**
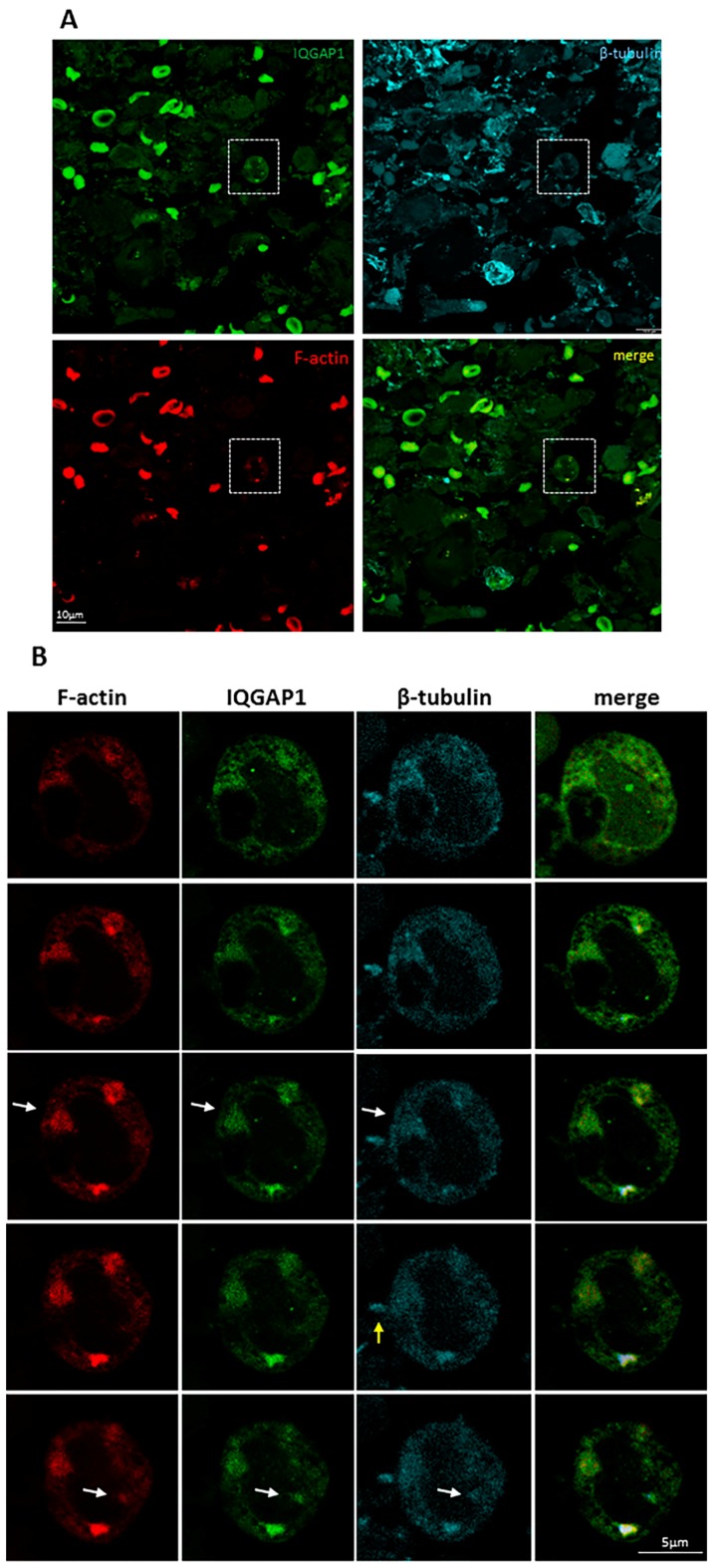
Triple immunofluorescence on GBM tissue section to detect F-actin (red), β-tubulin (cyan) and IQGAP1 (green) proteins: (**A**) confocal image resulting from the maximum projection of a z-series; and (**B**) sequencial confocal serial sections from the same z-serie focusing on the cell marked in (**A**). F-actin (first column), IQGAP1 (second column), β-tubulin (third column), merge (fourth column). Sequential sections revealed slight variations in the localization of the three proteins (white arrows). Yellow arrow: β-tubulin^+^ expression in the apical side of a cell protrusion. Bar in **A** is 10 µm; bar in **B** is 5 µm.

Taking into account that IQGAP1 and Iba1 proteins have recently been implicated in the formation of podosome-like structures [27,28], in order to assess the nature of these strong punctate and/or ring-shaped structures observed, GBM tissue sections were co-immunostained for IQGAP1 protein and the podosome/invadopodium markers F-actin and β-tubulin. Figure 4A shows the confocal image resulting from the maximum projection of a z-series in which F-actin, IQGAP1 and β-tubulin seemed to colocalize in such structures. However, a deeper analysis of the sequential sections revealed slight variations in the localization of the three proteins (Figure 4B, white arrows).

**Figure 5:**
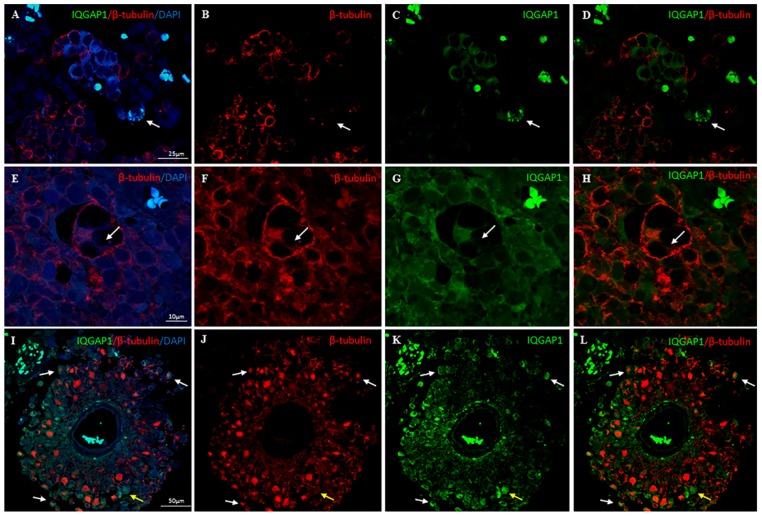
Double immunolocalization of IQGAP1 (green) and β-tubulin (red) on human GBM tissue sections. (**A**–**H**) colocalization of β-tubulin and IQGAP1 is detected in several cells at membrane ruffles, in cytosol and/or in the nuclear envelope; arrow in (**A**–**D**) points to a cell with podosome-like structrures highly positive for IQGAP1; and arrow in (**E**–**H**) points to a long cell protrusion where IQGAP1 and β-tubulin only colocalize at the basal side; (**I**–**L**) Typical GBM perivascular pseudorosette. Yellow arrow points to a cell with short podosome-like structures IQGAP1^+^/β-tubulin^+^ and a single longer protrusion highly positive for β-tubulin. White arrows show several cells co-expressing β-tubulin and IQGAP1. IQGAP1^+^ cells are also observed in the tunica intima and in tight contact with the tunica adventitia of the central vessel. Bar in **A**–**D** is 25 µm; bar in **E**–**H** is 10 µm; bar in **I**–**L** is 50 µm.

A colocalization of β-tubulin with F-actin and IQGAP1 was also observed in short cell protrusions, while in larger protrusions this colocalization often could only be observed at the basal pole of the structure; in the apical pole only β-tubulin was detected (Figure 5E–L). Moreover, in several cells, co-staining for β-tubulin and for IQGAP1 was detected at membrane ruffles, in cytosol and/or in the nuclear envelope (Figure 5).

**Figure 6:**
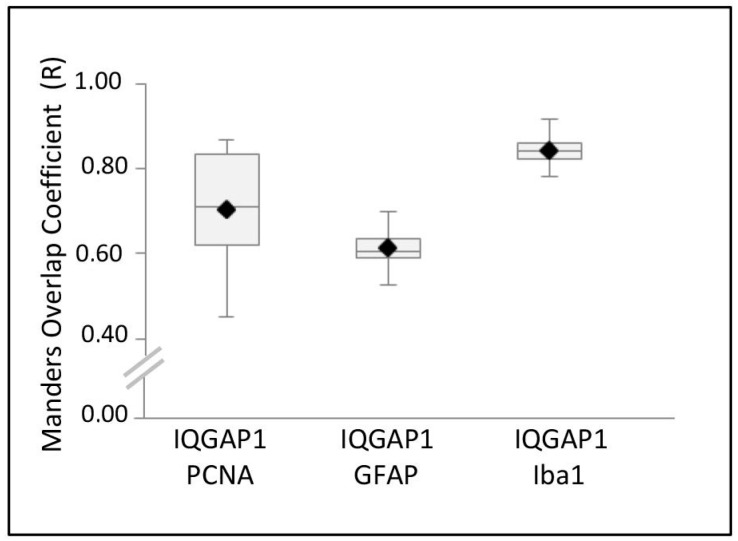
Box-and-whisker plot illustrating the results of the colocalization analyses of IQGAP1 protein with PCNA, GFAP or Iba1 in GBM, obtained using ImageJ Manders’ coefficients plug-in. Boxes indicate interquartile ranges, while whiskers indicate ranges of maximal and minimal values.

**Statistics:** Figure 6 shows a box-and-whisker plot illustrating the colocalization analyses of IQGAP1 protein with GFAP, PCNA or Iba1 obtained using the ImageJ Manders’ coefficients plug-in. The range value of Manders’ Overlap coefficient (*R*) is 0–1, with 0 representing low colocalization, and 1 high colocalization. Boxes indicate interquartile ranges, whiskers indicate ranges of maximal and minimal values. Supplementary Materials Table S1 shows the mean values and the standard deviations of R obtained from this analysis.

## 3. Discussion

GBM is constituted by following kind of cells: Glioma stem cells (GSCs), astrocytes, vascular cells (endothelial and pericytes) [29,30], remaining neurons and immune cells (GIMs or TAMs). The use of antibodies against cell- and function-specific markers allowed us to study the involvement of IQGAP1 in the onco-biology of GBM by observing its cellular and subcellular localization and the intensity of expression. IQGAP1 immunoreactivity is present in virtually all GBM cells, including GSCs, astrocytes, endothelial cells and GIMs or TAMs.

### 3.1. IQGAP1 and Mitosis

The variable intensity and subcellular localization of staining with anti-PCNA antibodies (Figure 1E–H) showed the asynchrony in cell division of GBM. PCNA is a DNA clamp that increases the processivity of DNA polymerase δ in eukaryotic cells [31]. Among a cloud of spread cells with high expression level of PCNA, cell clusters of lesser PCNA-IQGAP1 co-expression levels than surrounding tissue can be seen, as the one shown in Figure 1F. Strikingly, cells in these clusters possess higher content of IQGAP1 protein (Figure 1E,G,H).

After synthesis of PCNA in the cytoplasm of cells, PCNA then enters into the nucleus and S phase of mitosis begins. A role for IQGAP1 in regulating early S phase replication events has been proposed [32]. The authors observed that the nuclear IQGAP1 localization was low in asynchronous cells, but was significantly increased in cells arrested in G_1_/S phase, suggesting that IQGAP1 enters the nucleus at G_1_/S phase and exits in late S phase [32].

Figure 6 shows a mean of quotient PCNA/IQGAP1 of 0.677 (Supplementary Materials, Table S1), a high grade of mitosis-proliferation, as index of malignity of this tumor.

### 3.2. IQGAP1 in GBM Astrocytes and GSCs

Astrocytes maturity in GBM varies from GSC to well differentiated astrocytes. The prevalent population of cells, at variable intensities, is IQGAP1^+^/GFAP^+^ (Figure 2I–T and Figure 6; Supplementary Materials, Table S1). Our preparations showed that most IQGAP1^+^/GFAP^−^ cells are preferentially located within or around blood vessels (Figure 1M–T), confirming data reported by Balenci et al. [33]. Our labeling with specific antibodies identified cells around and within vessels as CSCs or GSCs (nestin^+^/IQGAP1^+^), Tumor Associated Macrophages (TAMs) and endothelial cells (CD31^+^/IQGAP1^+^), microglia (Iba1^+^/CD31^−^/IQGAP1^+^) and, in addition to these well characterized kind of cells, colonies of IQGAP1^+^/Iba1^+^/nestin^+^ cells are found surrounding nestin^+^ cells (Figure 2K–O). An increasing number of studies has recently provided evidences supporting the Hierarchical Cancer Stem Cell Model, which postulates that tumors are composed of biologically distinct cell classes with different functional abilities and behavior [24,25,26,34]. One of the hints of this model is the distinction between stem cells and progenitor cells, which differ in terms of hierarchy and biology. Stem cells are multipotent and with a high self-renewal capacity, while progenitor cells have a restricted differentiation potential and a limited self-renewal capacity. In this context, it has been observed that nestin protein is expressed not only in GSCs, but also further down in the stem cell hierarchy as it is expressed in more differentiated cells (progenitor cells) as well [25,26,34]. The observed IQGAP1^+^/Iba1^+^/nestin^+^ cells could represent a common ancestor for TAMs and GBM tumorigenic neural precursors and/or, probably, an interconvertible multipotent progenitor cell form of both kinds and/or glioblastoma amplifying cancer cells [33] (GSCs or progenitor cells). However, this hypothesis need to be further investigated.

### 3.3. IQGAP1 in Macrophages

GIM cells or TAMs can reach as much as 30% of total GBM mass and come from two origins: Resident macrophages (microglia) and circulating monocyte-derived macrophages [35,36,37,38], all implicated in GBM progression.

The morphology of Iba1^+^ cells (Figure 2M,R) ranged from ramified cells, like resting microglia, to bigger cells (20–25 µm long) with the characteristic appearance of activated microglia, which is correlated with proliferation [39] and, in addition, some often were nestin^+^ cells. The pro-inflammatory phenotype of the GBM associated microglial cell seems to be suppressed within the GBM environment becoming a cell that promotes glioma cell migration, neither oligodendrocytes nor endothelial cells promoted the migration and the effects of macrophages from the periphery on glioma growth are different from those of microglia [38]. TAMs were mainly located in the marginal area (Supplementary Materials, Figure S2), close to nestin^+^ GSCs and both around microvessels CD31^+^ endothelial cells (Figure 2F–O) confirming data reported by Ye et al. [40]. This fact, along with pericyte-endothelial interactions, favors pathological angiogenesis [30] exhibiting a highly invasive potential.

### 3.4. IQGAP1 in Neurons

Neurons in GBM are the scarcest population of cells, besides, neurons present a broad grade of differentiation status. Most Map2*^+^* cells, if not all, are IQGAP1^+^ stained (Figure 3C,D,K–L and Supplementary Materials, Figure S1), in addition, there is a population of Map2^+^/IQGAP1^+^/Iba1^+^ (Figure 3H). Map2^+^/IQGAP1^+^/Iba1^+^ cells could represent cells undergoing a kind of Epithelial—Mesenchymal Transition (EMT) that lead to a dedifferentiate status mimicking macrophage-like cells [41,42]. In a mouse model of CNS metastasis, tumor cells behave like macrophages within the vasculature and during extravasation, expressing GIM/TAM markers, Iba1 among them [42,43]. We assume that all neurons express IQGAP1 in a polarized manner, however, Map2^+^/IQGAP1^−^ neurons, such as those shown in Supplementary Materials Figure S1, do not have IQGAP1 staining because it is probably not in that part of the stained histology slice, but we assume that IQGAP1 protein is expressed somewhere else in that polarized cell. Recently, Zhou et al. [44] have reported that transcriptional upregulation of MAP2 in malignant glioma through PKA signal transducer and activator of transcription 3 (STAT3) pathways led to polymerization of tubulin ending in ossification of microtubule dynamics and reduction of glioma cell invasion.

### 3.5. IQGAP1 in Endothelial Cells

GBM presents high microvascular proliferation forming glomeruloid structures, probably overstimulated by the overexpression of the VEGF (vascular endothelial growth factor) and poor pericyte recruitment [30,36]. Endothelial cells express IQGAP1 and exhibit a high proliferation and migratory capacity and are highly resistant to apoptosis. In addition, transversal cut of small size vessels gives a typical perivascular pseudorosette conformed by all kind of cells mentioned in this study. An example is shown in Figure 5I–L, where IQGAP1^+^ cells are observed in the tunica intima and in tight contact with the tunica adventitia. Endothelial cells lead angiogenesis by developing endothelial podosome/invadopodia rosettes [45], critical in GBM progression.

### 3.6. IQGAP1 in Podosome/Invadopodia

**Figure 7:**
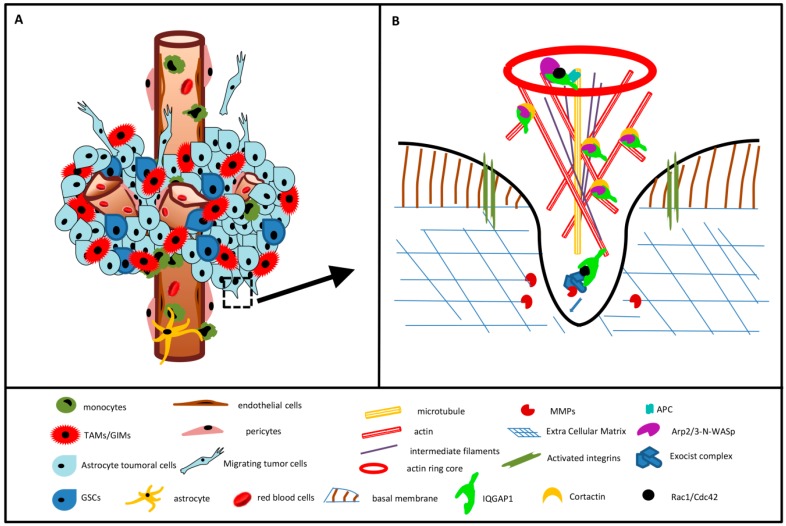
Glioblastoma multiforme and Invadopodia models. (**A**) GBM cellularity: Most GBM cell kinds, for different scopes, generate podosome/invadopodia structures; (**B**) Box in (**A**): IQGAP1 coordinates actin assembly and the exocytosis trafficking machinery in podosome/invadopodia formation.

With the only one exception of neurons, in our observation all described kind of cells in GBM present podosome/invadopodia-like structures. During tumor invasion, GBM cells from the tumor migrate towards the neighboring normal tissue by extending their edge actin-rich cancer-specific membrane protrusions forming invadopodia with the ability to infiltrate and degrade physical barriers, such as basement membranes, extracellular matrix (ECM), and cell junctions by metalloproteinases (MMPs) [30,35,36]; podosome/invadopodia are identified for their high expression levels of F-actin and/or cortactin [46]. The role of IQGAP1 as a scaffold protein in the delivering process of MMPs has been demonstrated in cell lines and animal models, as *C. elegans*, *zebrafish*, sea squirt, mice and rat [33,40,47,48]. Figure 7 is a simplified model of some of the proteins involved in the podosome/invadopodia generation mechanism and a model of the involvement of IQGAP1 in GBM progression based on the present study. Figure 7 also includes IQGAP1 relations with the upstream signaling pathway involving the small GTP-binding proteins Cdc42/Rac1 and Arp2/3-N-WASp interacting complexes [38,47,49].

### 3.7. Final Considerations and Future Research Directions

Despite of the different molecular pathogenesis and progression of gliomas giving rise to primary or secondary GBM [2], within the limits of our study we have not seen differences in the scattered cellular expression of IQGAP1 between them.

A recent study by Lu et al. [50] reported that expression of miR-124a is downregulated in glioma tissues and in human glioma cell lines and, furthermore, restoration of miR-124a levels or the knockdown of IQGAP1 inhibited glioma cell proliferation and invasion.

Our study shows that an involvement of IQGAP1 in the spatiotemporal organization and activation of structural and signaling molecules [10,11] takes place not only in healthy cells, but also in tumorigenic cells, specifically in GBM progression with different subcellular localizations and expression levels depending on the status of the cell within the tumor. Switching GBM to a successfully treatable entity will require a change from heterogeneous to a more homogeneous tumor status. The regulation of IQGAP1 as a synchronizer expressed in all kind of cells underlying GBM onco-biology could improve expectations for tumor invasion, resistance and recurrence. These wills pass necessarily through new studies directed to a better understanding of the molecular relationships among IQGAP1 and its partner proteins in order to block specific functions related to tumorigenesis.

## 4. Materials and Methods

### 4.1. Patients and Tumor Tissue

Clinical and Pathology data were collected from 39 patients, 33 primary GBM (14 males and 19 females) and 6 secondary GBM (5 males and 1 female). The study was approved by the Ethics Committee of La Laguna University (La Laguna, Canary Islands, Spain) and the Ethical Committee of Nuestra Señora de Candelaria University Hospital (HUNSC); Santa Cruz de Tenerife, Canary Islands, Spain (no. 198/2008, approved on 16 September 2008). All patients have been treated in the HUNSC between years 2007 and 2015 and provided informed consent for the diagnosis and research of tissue specimens prior to enrolment in the study. GBM samples were taken after initial surgery in the HUNSC before patients received radiation or chemotherapy. Paraffin-embedded tissue samples and corresponding clinical data were used ensuring patient’s anonymity.

### 4.2. Antibodies

Primary antibodies: Rabbit anti-human polyclonal antibody against IQGAP1 (dilution 1:250; #ABT186 EMD Millipore, Billerica, MA, USA); mouse monoclonal antibody clone PC10 against anti-Proliferating Cell Nuclear Antigen (PCNA) (dilution, 1:100; #1486772 Roche Diagnostics GmbH, Mannheim, Germany); mouse monoclonal anti-human cluster of differentiation (CD)31 (ready-to-use; #IR610 Dako, Glostrup, Denmark); mouse monoclonal anti-β-tubulin (dilution, 1:150; #sc-101527 Santa Cruz Biotechnology Inc., Dallas, TX, USA); mouse monoclonal antibody against human nestin (dilution 1:25; #MAB1259 R&D Systems, Minneapolis, MN, USA); goat polyclonal antibody against Iba1 (dilution 1:500; #ab107159 Abcam, Cambridge, UK); mouse monoclonal anti-Glial Fibrillar Acidic Protein (GFAP) (dilution 1:100; #G3896 Sigma, Saint Louis, MO, USA); mouse monoclonal anti-Microtubule Associated Protein-2 (MAP2) (dilution 1:500; #MAB378 Chemicon International, Temecula, CA, USA). Secondary antibodies: fluorescein isothiocyanate (FITC)-conjugated goat pAb against rabbit IgG (dilution 1:200; #F9887; Sigma‑Aldrich, St. Louis, MO, USA); goat pAb against mouse IgG DyLight^®^ 650 (dilution 1:100; #ab97018; Abcam); Cy™3 AffiniPure Donkey Anti-Goat IgG (H+L) (dilution 1:400; #705-165-147 Jackson Immunoresearch Laboratories, West Grove, PA, USA).

### 4.3. Image Analysis and Statistical Analysis

To compile tables, two independent observers evaluated the specimens blindly. Staining intensities were graded as absent (−), weak (+), moderate (++) or strong (+++). These cut-offs were established by consensus between each investigator following an initial survey of the entire blind-coded material. In cases where scorings differed by more than one unit, the observers re-evaluated the specimens to reach a consensus. In other cases, means of the scorings were calculated.

### 4.4. Colocalization Analysis

For red/green colocalization analysis, the open resource digital image analysis software *ImageJ* has been used (Rasband, W.S., ImageJ, National Institutes of Health, Bethesda, MD, USA, http://rsb.info.nih.gov/ij/, 1997–2004), implemented with the Manders’ coefficients plug-in developed by Tony Collins (Wright Cell Imaging Facility, Toronto, ON, Canada), which calculates Manders’ coefficients for two 8- or 16-bit images or stacks. Briefly, all confocal images were captured at the same magnification (40×) and with the same levels of contrast and brightness. Using the ImageJ tools, images were then converted in 8-bit grey scale, background subtracted and analyzed for colocalization with “Manders’ coefficients” plug-in. The range value of Manders’ Overlap coefficient (*R*) is 0–1, with 0 representing low colocalization, and 1 high colocalization. The values obtained were used to create a box and whisker plot to visualize the results. In Supplementary Materials Table S1 are reported the mean and standard deviation of the values obtained.

### 4.5. Double/Triple Immunofluorescence Simultaneous Staining

Immunofluorescent staining of 10% formalin-fixed paraffin-embedded tissue sections was performed as previously described [51]. Briefly, 5 μm-thick tissue sections were deparaffinized in xylene and hydrated in a graded series of alcohol baths. Heat-induced epitope retrieval was achieved by heating samples in sodium citrate buffer (pH 6.0) at 120 °C for 10 min in an autoclave. Once non-specific sites were blocked with 5% bovine serum albumin or normal donkey serum in Tris-buffered saline (TBS) for 1 h at room temperature, tissue sections were then incubated simultaneously with a mixture of two/three distinct primary antibodies (rabbit against human target 1, mouse against human target 2, goat against human target 3) overnight at 4 °C. Slides were then incubated for 1 h at room temperature in the dark with a mixture of two/three secondary antibodies raised in different species and conjugated to different fluorochromes. For actin staining, tissue sections were incubated in the dark for 1 h at room temperature with Phalloidin- tetramethylrhodamine B isothiocyanate (Phalloidin-TRITC) (dilution 1:500; #sc-301530 Santa Cruz Biotechnologies, Dallas, TX, USA). Slides were mounted with ProLong^®^Diamond Anti-fade Mountant with DAPI (Molecular Probes^®^; Thermo Fisher Scientific, Inc., Waltham, MA, USA) to visualize cell nuclei. Slides were analyzed using Olympus FV1000 (Olympus Corporation, Tokyo, Japan) and Leica SP8 (Leica Microsystems, Wetzlar, Germany) confocal microscopes.

## Figures and Tables

**Table 1:** Markers used in this study.

Antibody	Marker of
*Anti-GFAP*	Astrocytes
*Anti-Iba1*	Microglia/Macrophages
*Anti-PCNA*	Proliferative cells
*Anti-Nestin*	Neural stem cells/Glioblastoma stem cells
*Anti-CD31*	Endothelial cells/Monocyte derived macrophages
*Anti-Map2*	Mature neurons
*Anti-β-tubulin*	Microtubules
*Phalloidin-TRITC*	F-actin

**Table 2:** Protein expression levels and localization of IQGAP1 in primary and secondary GBMs.

Primary GBM	Patient 1	Patient 2	Patient 3	Patient 4
Intensity localization	+++/++ m,n,c	+++/++ m	+++/++ m,n	+/− m,c
**Secondary GBM**	**Patient 5**	**Patient 6**	**Patient 7**	
Intensity localization	+++ m,n	+++/− m,c	+++/++ m,n,c	

+/−, variable faint/none; +++/++, variable high/medium; +++/−, variable high/none; +++, high; m = membrane; c = cytoplasm; n = nucleus and/or nuclear envelope.

## Supplementary Materials

Supplementary materials can be found at www.mdpi.com/1422-0067/18/1/150/s1.

## Abbreviations

ECM	Extracellular matrix
EMT	Epithelial-mesenchymal transition
FITC	Fluorescein isothiocyanate
DAPI	Diamond Anti-fade Mountant
GBM	Glioblastoma multiforme
GIMs	Glioma-infiltrating myeloid cells
Iba1	Ionized calcium binding adaptor molecule 1
IQGAP1	IQ motif containing GTPase-activating protein
MAP2	Microtubule-associated protein 2
PCNA	Proliferating cell nuclear antigen
TAMs	Tumor associated macrophages
TRITC	Tetramethylrhodamine B isothiocyanate
