# Supplementary material for: IQGAP1 in Podosomes/Invadosomes Is Involved in the Progression of Glioblastoma Multiforme Depending on the Tumor Status
Source: Int J Mol Sci. 2017 Jan 13;18(1):150. doi: 10.3390/ijms18010150 (PMC5297783; doi:10.3390/ijms18010150)
Supplement: Supplementary file 1 [file ijms-18-00150-s001.pdf]

Supplementary Materials: IQGAP1 in Podosomes/Invadosomes Is Involved in the Progression of Glioblastoma Multiforme Depending on the Tumor Status

Deborah Rotoli, Natalia Dolores Pérez-Rodríguez, Manuel Morales, María del Carmen Maeso, Julio Ávila, Ali Mobasheri and Pablo Martín-Vasallo

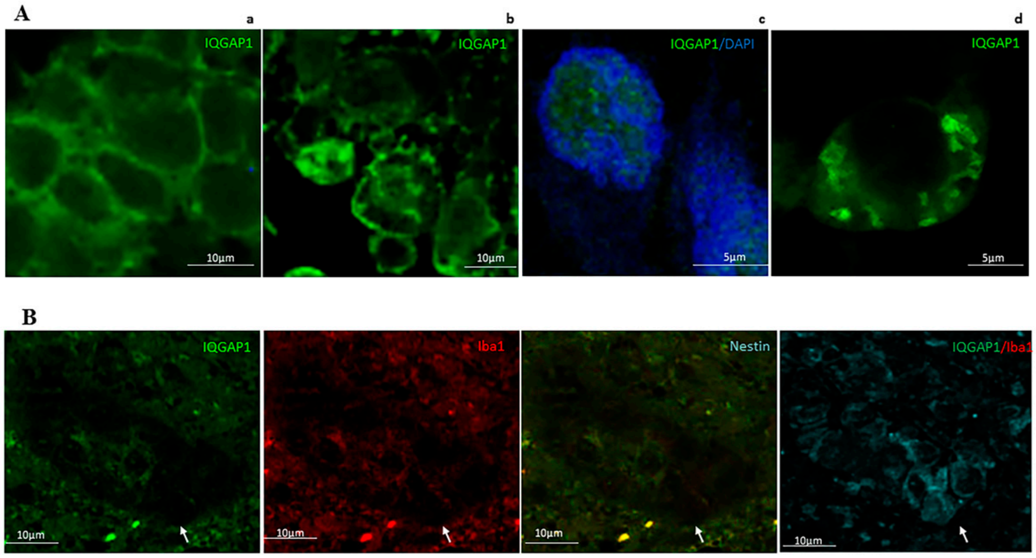

Figure S1. (**A**) Higher magnification of Figure 1A–D. IQGAP1 protein in GBM detected in: (**a**) plasma membrane; (**b**) cell protrusions, plasma membrane and cytosol; (**c**) nucleus; and (**d**) cell with podosome-like structures highly positive for IQGAP1; (**B**) Higher magnification of Figure 2K–O. Triple immunolocalization of IQGAP1 protein (green), Iba1 (red) and nestin (cyan). Arrow points to a nestin^{+}/IQGAP1^{-}/Iba1^{-} cell flanked by nestin^{+}/IQGAP1^{+}/Iba1^{+} cells.

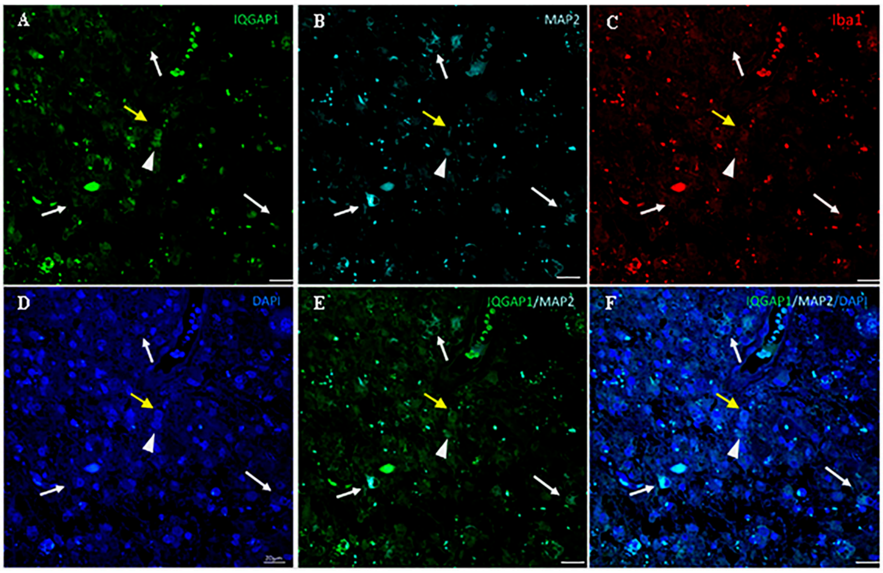

Figure S2. Triple immunolocalization of IQGAP1 protein (green), the microglia/macrophage marker Iba1 (red) and the Microtubule-associated protein 2 (MAP2, cyan) in GBM tissue sections. (**A**) IQGAP1; (**B**) MAP2; (**C**) Iba1; (**D**) DAPI; (**E**) IQGAP1/MAP2 merge; (**F**) IQGAP1/MAP2/DAPI merge. White arrows point to Map2+ neurons IQGAP1−/Iba1−. Yellow arrows points to a cell IQGAP1+/Iba1+. Arrowhead points a cell IQGAP1+/nestin+. Bar 20 µm.

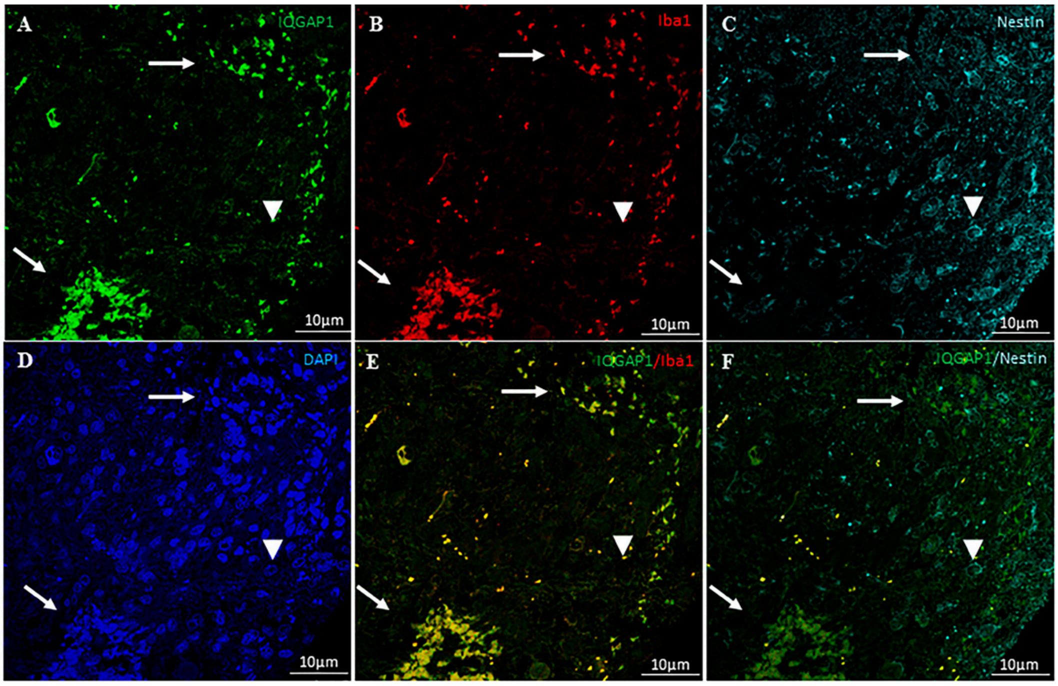

Figure S3. Triple immunolocalization of IQGAP1 protein (green), the microglia/macrophage marker Iba1 (red) and the CSC marker nestin (cyan). (**A**) IQGAP1; (**B**) Iba1; (**C**) Nestin; (**D**) DAPI; (**E**) IQGAP1/Iba1 merge; (**F**) IQGAP1/Nestin merge. In this highly vascularized area of a GBM tissue section, many nestin+ cells are observed (arrowhead). Note the massive presence of macrophages (Iba1+/IQGAP1+/nestin-) in the periphery (arrows). Scale bar: 10 µm.

Table S1. IQGAP1/PCNA, IQGAP1/GFAP and IQGAP1/Iba1 colocalization. Mean and standard deviation of values obtained with the ImageJ plug-in "Mander's coefficients".

	n	*R*	**Ch1/Ch2**	**M1**	**M2**	**Ch1 Thresh, Ch2 Thresh**
**Ch1 = GFAP Ch2 = IQGAP1**	27	0.59 ± 0.05	0.995 ± 0.02	0.969 ± 0.02	0.971 ± 0.01	1;255
**Ch1 = PCNA Ch2 = IQGAP1**	20	0.677 ± 0.13	0.750 ± 0.29	0.851 ± 0.14	0.739 ± 0.20	1;255
**Ch1 = Iba1 Ch2 = IQGAP1**	14	0.84 ± 0.04	0.801 ± 0.15	0.954 ± 0.04	0.835 ± 0.09	1;255

n = samples analyzed. *R* = Mander's Overlap coefficient. Value Range: 0–1, with 0 = low colocalization and 1 = high colocalization; Ch1/Ch2 = red/green pixel ratio. Ratio ≈ 1 allows the use of Mander's Overlap coefficient (R); M1, M2 = Mander's Colocalization coefficients for channel 1 (M1) and channel 2 (M2). Value range: 0–1, with 0 = no colocalization 1 = all pixels colocalize; Ch1 thresh, Ch2 thresh = ch1 and ch2 threshold.
